# Embodying cognitive ethology

**DOI:** 10.1177/09593543221126165

**Published:** 2022-10-11

**Authors:** Helen L. Ma, Michael R. W. Dawson, Ruby S. Prinsen, Dana A. Hayward

**Affiliations:** University of Alberta

**Keywords:** attention, cognition, cognitive ethology, embodied cognition, real-world environment

## Abstract

Cognitive psychology considers the environment as providing information, not affecting fundamental information processes. Thus, cognitive psychology’s traditional paradigms study responses to precisely timed stimuli in controlled environments. However, new research demonstrates the environment does influence cognitive processes and offers cognitive psychology new methods. The authors examine one such proposal: cognitive ethology. Cognitive ethology improves cognitive psychology’s ecological validity through first drawing inspiration from robust phenomena in the real world, then moving into the lab to test those phenomena. To support such methods, cognitive ethologists appeal to embodied cognition, or 4E cognition, for its rich relationships between agents and environments. However, the authors note while cognitive ethology focuses on new methods (epistemology) inspired by embodied cognition, it preserves most traditional assumptions about cognitive processes (ontology). But embodied cognition—particularly its radical variants—also provides strong ontological challenges to cognitive psychology, which work against cognitive ethology. The authors argue cognitive ethology should align with the ontology of less radical embodied cognition, which produces epistemological implications, offering alternative methodologies. For example, cognitive ethology can explore differences between real-world and lab studies to fully understand how cognition depends on environments.

Psychological schools are defined by both an ontology (the core topics studied) and an epistemology (the methods used). As a result, ontological and epistemological pressures cause schools to change. Alternative schools emerge when new proposals replace an older school’s core assumptions and methodologies.

Behaviorism’s rise illustrates how such pressures change psychology. Watson (1913) challenged existing ontology by arguing psychology must explain behavior, not consciousness. Watson challenged existing epistemology by abandoning a core method—introspection—and replacing it with behavioral observations.

Ontological and epistemological pressures also incite changes within a school. Consider a famous critique of behaviorism (Breland & Breland, 1961). Breland and Breland (1961) described many failures to train animals via core behaviorist paradigms. Instead of learning desired behaviors, animals developed responses could only be explained by appealing to the animals’ instincts. Breland and Breland (1961) concluded “the behaviour of any species cannot be adequately understood, predicted, or controlled without knowledge of its instinctive patterns, evolutionary history, and ecological niche” (p. 684).

Breland and Breland (1961) provided a methodological critique by questioning behaviorism’s ecological validity: whether lab results generalize to the world (Bem & Funder, 1978; Bem & Lord, 1979; Hovland, 1959). However, they also offered ontological concerns. They challenged researchers who assumed lab studies of animal learning can ignore animal history or species differences. The second challenge questioned the assumption all actions can be equally conditioned.

Breland and Breland’s (1961) critique arrived at the same time as other external pressures produced a new psychological school: cognitivism (Gardner, 1984). To cognitive psychologists, behaviorist theory trapped passive agents in a sense–act cycle. In this cycle, stimuli directly cause responses. Cognitivism offered a new ontology in which active agents only responded after processing sensed information—replacing sense–act processing with a sense–think–act cycle (Dawson, 2013; Hurley, 2001). In the sense–think–act cycle, sensing does not directly cause acting. Instead, thinking—cognitive processing—always intervenes (see Figure 1 [A]). Cognitive psychologists view thinking as the rule-governed manipulation of mental representation and believe cognition’s purpose is to plan action. The corollary of this belief is action cannot occur without planning.

**Figure 1.**
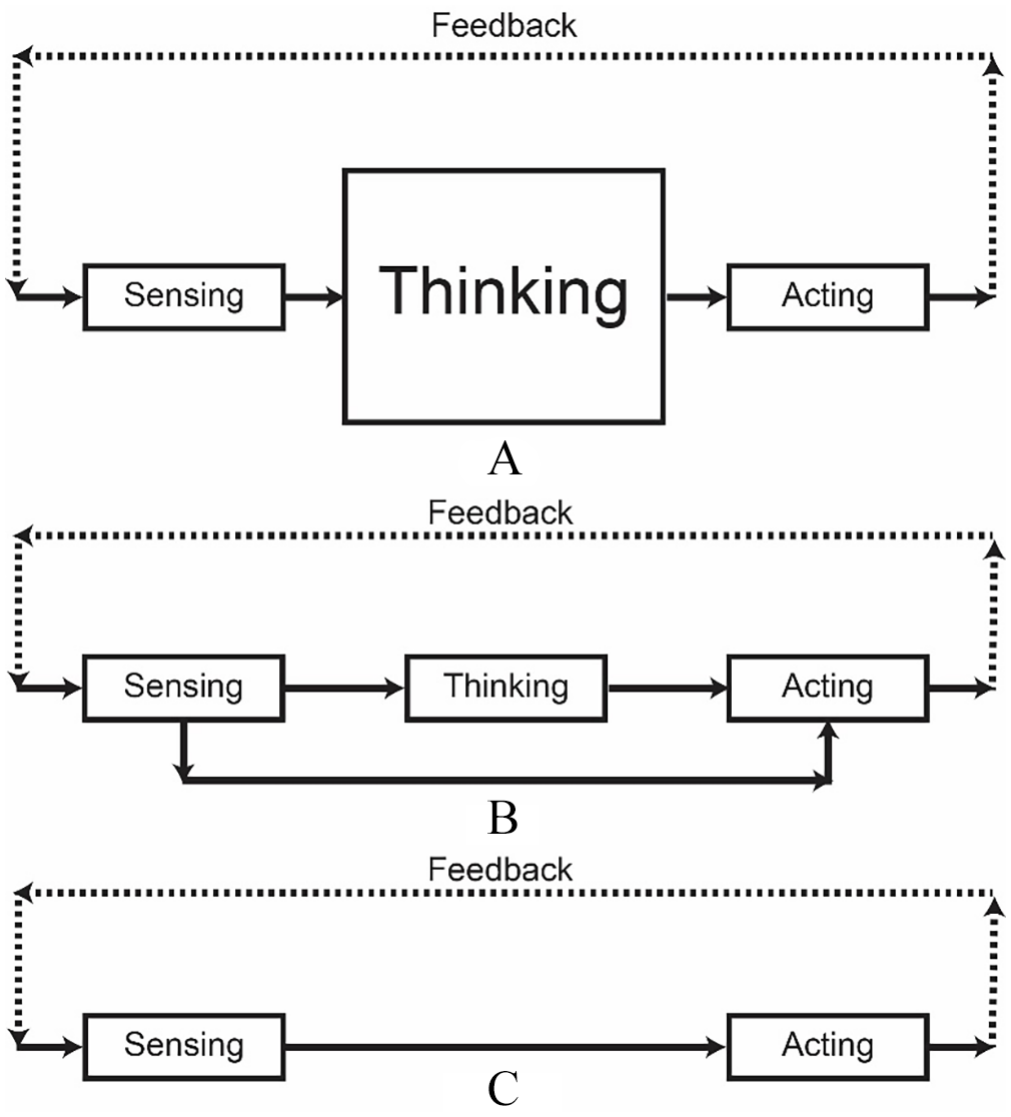
Theories of Cognition. With the rise of embodied cognition, theories of cognition can be placed on a continuum from pure sense–think–act processing to pure sense–act processing. The pure sense–think–act (A) cycle characterizes cognitive psychology’s traditional view of processing. Thinking necessarily mediates the relationship between sensing and acting. Action on the world can change the information available for sensing, as indicated by the feedback loop illustrated with the dashed arrows. The “thinking” function is larger than the other two because cognitive psychologists overemphasize representational processing and underemphasize both sensing and acting (J. R. Anderson, 1983; Newell, 1990). Less radical embodied cognitive psychologists propose cognition involves both sense–think–act processing and sense–act processing (B). Some processing involves using representational processes to mediate relationships between sensed information and action on the world. Some processing permits direct links between sensing and acting without the need for thinking or planning. The pure sense–act (C) cycle is endorsed by embodied cognitive psychologists who reject the sense–think–act cycle. In the sense–act cycle, representational processes (thinking) have disappeared. Sensing is linked directly to acting, and complex behavior emerges from feedback between the two functions. We propose cognitive ethology would be well served by endorsing processing is represented somewhere in the middle of the continuum (e.g., more like B and less like A or C).

The sense–think–act cycle’s ontological pressure was accompanied by novel methodologies, including computer simulations of cognition (Feigenbaum & Feldman, 1963; Newell et al., 1958; Newell & Simon, 1972) and a functionalist philosophy of science (Cummins, 1983; Fodor, 1968). Cognitive psychology cast itself as both more complex and more nuanced than behaviorism, and currently dominates modern experimental psychology (Glenberg et al., 2013).

However, like behaviorism, cognitive psychology is subject to internal pressures (e.g., see Breland & Breland, 1961). Concerns about cognitive psychology’s ecological validity are leading to proposals of new methodologies. This article explores one proposal, which Kingstone and his colleagues call *cognitive ethology* (Kingstone, 2020; Kingstone et al., 2008; Smilek et al., 2006). We focus on Kingstone’s notion of cognitive ethology because it aims to improve the ecological validity of cognitive psychology by appealing to core ideas in embodied cognition, which we describe below. We describe the ontological and epistemological pressures cognitive ethologists use to improve cognitive psychology. However, we also show such pressures can lead cognitive ethology to more radical ontological positions, moving it far away from traditional cognitivism. We argue cognitive ethologists must consider the implications of radical ontologies when crafting better methodologies for cognitive psychology.

## From cognitivism to cognitive ethology

Ontological commitments accompany cognitivism’s sense–think–act cycle. By assuming cognition involves a sense–think–act cycle, cognitive psychologists can study cognition independently from the world. In the sense–think–act cycle, the world merely provides information. As a result, cognitive psychologists often develop cognitive theories ignore both sensing and acting (Newell, 1990). Cognitive psychologists are less concerned about sensing and acting because they place thinking at the core of the sense–think–act cycle. To cognitive psychologists, cognitive theory must explain thinking—actively processing, representing, reorganizing, and supplementing information received from the world—because thinking, not the world, is responsible for causing action. Cognitivists study mental representations of the world, ignoring the world itself.

The ontological commitments of the sense–think–act cycle also carry epistemological commitments. Because they assume that sensing merely provides the elements required to construct representations, cognitive psychologists expect such elements will produce the same representations in both the lab and more natural settings. Presumably, cognitive processes are common to all individuals and all settings. Kingstone et al. (2008) call this the *invariance assumption*. The invariance assumption permits cognitive psychologists to assume lab studies are sufficient to identify, study, and explain core cognitive processes.

In addition, Kingstone et al. (2008) note the invariance assumption is typically coupled with the *assumption of control*. Within the lab setting, the assumption of control encourages researchers to reduce environmental variation, permitting changes in behavior to be attributed to cognitive processes rather than stimuli. Kingstone et al. (2008) argue the invariance assumption and the assumption of control define cognitive psychology’s methodology:

The assumption of process stability enables the scientist to be concerned with real-life situations without ever having to leave the lab. In addition, the assumption of control drives the scientist increasingly away from complex real-life situations to paradigms that are simple, contrived, and artificial. (p. 319)

Ironically, research practices adopting the two assumptions make cognitive psychology open to criticisms like those Breland and Breland (1961) directed towards behaviorism. Cognitive psychology has long faced concerns about its ecological validity (Broadbent, 1993; Neisser, 1976; Norman, 1980; Winograd & Flores, 1987). In fact, Kingstone et al. (2008) propose a new methodology, which they call *cognitive ethology*, for increasing cognitive psychology’s ecological validity by rejecting cognitive psychology’s assumptions of invariance and control.

Cognitive ethology criticizes cognitive psychology for being overly dedicated to both lab-based studies and lab-dependent phenomena. As a result, Kingstone et al. (2008) argue cognitive psychology neglects its true goal—understanding how human cognition operates in the world. Cognitive ethology proposes a new epistemology to permit cognitive psychology to better understand real-world cognition.

Cognitive ethology’s new epistemology arises from a new ontology: cognitive ethologists reject the assumptions of invariance and control (Kingstone et al., 2008). Rather than assuming invariance, Kingstone et al. (2008) assume “processes may be contextualized to the situation within which they occur” (p. 321). Thus, cognitive ethologists expect cognition in the lab and in the world to differ. Rather than controlling environmental variance, Kingstone et al. (2008) argue for embracing and exploring such variance, for it may reveal key characteristics of cognition. Cognitive ethology’s altered assumptions produce a new paradigm: cognitive psychologists should first explore cognition in its natural setting. The purpose of studying real-world cognition is to generate hypotheses, which are further tested in a second phase of lab research. Cognitive ethologists study cognition “in the wild” first to guide and inform subsequent lab research.

Kingstone et al. (2008) use a pair of studies of human driving to illustrate cognitive ethology’s methodology. In the first, the steering-wheel angle and gaze direction were measured while participants drove an actual car along a real, demanding route (Land & Lee, 1994). Land and Lee (1994) discovered drivers, when encountering a bend in the road, focus on a tangent point that predicts the road curvature after being combined with the car’s head. In the second study, lab studies using a driving simulator examined how dynamic cues are used at different driving speeds (Land & Horwood, 1995). These two studies illustrate cognitive ethology’s logic because the first real-world study was required to identify cues and regularities for later study in the lab.

Before we proceed, it is important to note that while we focus on Kingstone’s (Kingstone et al., 2008) cognitive ethology, the term *cognitive ethology* also names other important research programs. Donald R. Griffin is the father of a different cognitive ethology which ascribes mental states to animals and uses mental states to explain animal behavior (Allen & Bekoff, 1995, 1997; Bekoff & Allen, 1992; Bekoff et al., 2002; Griffin, 1978, 1981; Griffin & Ristau, 1991; Ristau, 2013; Vauclair, 1997). Griffin (1978) reacted against what he called the “behavioristic taboo,” which excluded mental experience from scientific psychology. In other words, Griffin’s cognitive ethology shifted comparative psychology from behaviorism to cognitivism. In the context of Figure 1, Griffin’s cognitive ethology moves comparative psychology from radical sense–act (C) theories towards sense–think–act (A) theories. In contrast, Kingstone’s cognitive ethology moves the theories of cognitive psychology in the opposite direction (from A towards C).

While we recognize different cognitive ethologies exist, the current article focuses on the version proposed by Kingstone and his colleagues (Kingstone, 2020; Kingstone et al., 2008; Smilek et al., 2006). We focus on Kingstone’s cognitive ethology because we are interested in how cognitive theories change when concepts from embodied cognition are introduced (Figure 1). However, later, we briefly consider how ideas which emerge from our exploration of Kingstone’s cognitive ethology may also apply to variations of Griffin’s cognitive ethology.

## Cognitive ethology and the sense–think–act cycle

Kingstone et al. (2008) motivate their version of cognitive ethology by replacing two of cognitive psychology’s core assumptions. However, they preserve another core assumption—the sense–think–act cycle. Consistent with the sense–think–act cycle, cognitive ethologists abandon the invariance assumption to acknowledge different environments lead to different behaviors, but they assume different behaviors arise from changes in thinking or cognitive processing. For instance, Kingstone et al. (2008) attribute situational effects to changes in strategies or representations and underlying brain configurations: “cognitive processes change with situational context” (p. 319). Different situations presumably elicit different beliefs or goals. Beliefs and goals are intentional states, characteristic of thinking in the sense–think–act cycle (e.g., Dawson, 2013, Chapter 3). Cognitive ethology thus continues to view cognition as the rule-governed manipulation of mental representations and proposes different environments cause changes in how such manipulation proceeds.

By preserving the sense–think–act cycle, cognitive ethologists can view real-world investigations and lab studies as being complementary. Both study the “thinking” inside the cycle but do so from different perspectives. For Kingstone et al. (2008), lab studies offer subpersonal levels of explanation—accounts of core cognitive processes. Kingstone et al. (2008) contrast subpersonal levels of explanation with personal levels of explanation, which treat the person as a whole organism interacting with an environment. Personal-level explanations focus on situationally dependent subjective experiences, goals, and beliefs.

Cognitive ethologists believe that data supporting personal-level explanations identify situations and variables requiring further investigation in the lab:

Cognitive processes . . . cannot be fully understood at the subpersonal level unless the explanation is grounded in a personal-level understanding of peoples’ overt cognitive behaviour and their experiences, beliefs, and intentions as they select information in their everyday environments. (Kingstone et al., 2008, p. 329)

Such grounding requires personal and subpersonal approaches to assume sense–think–act processing. However, and as we discuss in the next section, cognitive ethology need not assume the sense–think–act cycle; rather, cognitive ethology could easily endorse sense–act processing.

## Cognitive ethology and embodied cognition

Cognitive ethology requires initial real-world studies of cognition to guide subsequent lab research. Real-world studies aim to provide personal-level explanations, which depend on the rich interactions between agents and environments. “Important aspects of cognition will only emerge when embodied individuals are considered as part of a system that involves their natural environment (including other individuals)” (Kingstone et al., 2008, p. 332). The renewed call to investigate cognition “in the wild,” along with other important factors outside the scope of the current article (Awh et al., 2012; Hayward & Ristic, 2017; McCrackin & Itier, 2018), has led to a large increase in the number of investigations in naturalistic settings, which often look a lot like cognitive ethology (Foulsham & Kingstone, 2017; Foulsham et al., 2011; Gregory & Antolin, 2019; Hessels et al., 2019; Macdonald & Tatler, 2018; Murphy & Murphy, 2018).

For example, to determine where people look in natural environments, Foulsham et al. (2011) first asked participants to walk across a campus and purchase a coffee before returning to the lab, all whilst wearing an eye tracker and a camera to record the environment. A week later, the same participants returned to the lab to watch video clips from their walk and other participants’ walks, and their gaze behaviors were compared. Overall, the gaze behaviors were similar. However, the participants in the lab were more likely to look at approaching pedestrians close to the walker compared to the participants in the natural environment, highlighting the importance of the environment when assessing cognition.

In focusing attention on embodied individuals in natural environments, cognitive ethologists align themselves with another approach to cognition, *embodied cognition* (Calvo & Gomila, 2008; Chemero, 2009; Clark, 1997, 2003, 2008, 2016; Dawson et al., 2010; Newen et al., 2018; Rowlands, 2010; Shapiro, 2014, 2019; Varela et al., 1991), which falls within a broader conception—namely, *4E cognition* (for embodied, embedded, extended, and enactive cognition, see Newen et al., 2018). Indeed, Kingstone et al. (2008) make the similarity between cognitive ethology and embodied cognition clear when making the case for cognitive ethology by citing core works (Gibson, 1966, 1979; Neisser, 1976) which others cite (Dawson, 2013; Shapiro, 2014; Wilson, 2002) as prototypical examples of embodied cognition.

Shapiro (2019) uses three ontological themes to characterize embodied cognition. According to the *conceptualization theme*, an agent’s concepts are constrained by the physical nature of its body. According to the *replacement theme*, interactions between an agent’s body and the world replace the agent’s need for mental representations. And according to the *constitution theme*, the body and world are literally parts of cognition and do not merely have causal effects on cognition.

Shapiro (2019) employs these three ontological themes because he believes embodied cognition is not a unified conception of the mind but is instead a research program which “exhibits much greater latitude in subject matter, ontological commitment, and methodology than does standard cognitive science” (p. 3). For example, Shapiro describes three different approaches (constitution, replacement, and conceptualization), which are all prototypical examples of embodied cognition but markedly differ from—and in many cases contradict—one another (Clark, 2008; Thelen et al., 2001; Varela et al., 1991). Shapiro’s position is that each approach emphasizes one of his themes over the other two, causing these differences (Clark [2008] emphasizes constitution; Thelen et al. [2001] emphasize replacement; Varela et al. [1991] emphasize conceptualization). Thus, for Shapiro, different embodied cognition programs emerge, all of which can be related to his ontological themes but can differ from one another in terms of their emphasis of each theme.

In our view, Figure 1 represents a broader ontological view which follows from Shapiro’s (2019) three themes, in which any combination of the themes moves a cognitive theory from A towards B. Regardless of the specific emphases of Shapiro’s three themes, these themes cause embodied cognition to react against cognitive psychology’s traditional view that thinking is the rule-governed manipulation of mental representations. Instead of viewing cognition as thinking and planning, embodied cognition views cognition as acting upon the world. It rejects the sense–think–act (A) cycle and instead assumes either pure sense–act (C) processing (M. L. Anderson et al., 2012; Barrett, 2011; Chemero, 2000, 2009; de Oliveira et al., 2019) or some hybrid (B) which includes both sense–think–act (A) and sense–act (C) processes (Clark, 1997, 2008; Dawson et al., 2010; Risko & Gilbert, 2016; Risko et al., 2016). An embodied theory which adopts stronger and stronger versions of Shapiro’s themes becomes an extreme anti-representational theory (like C).

By reacting against the sense–think–act cycle, embodied cognition dissociates itself from traditional cognitive psychology. First, rather than assuming changes in behavior reflect differences in underlying cognition (e.g., strategy changes or altered intentional states), embodied cognition explains such changes via direct appeals to the environment—highlighting the environment as a proper constituent of cognition. One famous example is the parable of the ant, in which Simon (1969) explains an ant’s complicated route along a beach: “Viewed as a geometric figure, the ant’s path is irregular, complex, hard to describe. But its complexity is really a complexity in the surface of the beach, not a complexity in the ant” (p. 24). When explaining the ant’s actions through the lens of “thinking,” one draws a very different conclusion from when explaining its actions through the lens of the “environment.” Other ideas for moving explanations from inside agents to outside worlds include affordances (Gibson, 1979), the *Umwelt* (Uexküll, 2001), and stigmergy (Grasse, 1959; Theraulaz & Bonabeau, 1999).

Second, rejecting the sense–think–act cycle removes mental representations from cognitive theory. One consequence of Shapiro’s (2019) replacement theme is “cognition can be explained without appeal to computational processes or representational states” (p. 5). Some versions of embodied cognition are radically antirepresentational (Chemero, 2000, 2009). However, we believe cognitive ethology can appeal to less radically antirepresentational versions of embodied cognitive science, which we discuss below (Clark, 1997, 2007).

Third, rejecting the sense–think–act cycle brings into question whether the lab is an appropriate setting for explaining cognition. If the environment is a constituent of cognition, one cannot study cognition in the lab unless one can replicate both the environment and the means used by the agent to act upon it. Embodied cognition’s three themes imply changing the world changes the mind. For this reason, classic examples of embodied cognition research take place in the world, not in the lab (Hutchins, 1995; Scribner & Tobach, 1997). More modern research continues this tradition. Examples include exploring a core representational concept (metaphoricity) by using in-depth analyses of real-world social interactions (Jensen & Cuffari, 2014), or investigating dynamic touch and object recognition via actions on unseen objects (Travieso et al., 2020). More generally, sense–act processing is central to topics like enactive perception (Noë, 2004, 2009, 2015), social interactions (Breazeal et al., 2009, 2016; Goldman, 2006; Goldman & de Vignemont, 2009), and the human-centered design of everyday objects (Dourish, 2001; Norman, 2002).

The consequences of embodied cognition rejecting sense–think–act processing have strong implications, not only for cognition but also for cognitive ethology. If cognitive ethology and embodied cognition are strongly aligned in the need to study cognition in the real world, then cognitive ethology’s proposed methodology becomes less secure because cognitive ethology’s methodology requires cognition to be grounded in the sense–think–act cycle. This changes the interpretation of these lab and real-world experiments, assuming that (a) sense–think–act processing permits cognitive ethology to view situational effects in terms of changing intentional states or cognitive strategies; (b) sense–think–act processing allows cognitive ethology to expect real-world studies to produce results similar to results for properly motivated lab studies; and (c) sense–think–act processing enables cognitive ethology to view real-world studies and lab experiments as complementary to one another. However, if cognition involves sense–act processing, such complementarity vanishes because real worlds differ substantially from controlled laboratories.

In the following section, we argue that cognitive ethology can endorse embodied cognition while pursuing research that combines real-world and lab investigations. However, to do so requires a more careful consideration of the relationship between cognitive ethology and embodied cognition. When carefully considering the relationship between the two, potential changes emerge in cognitive ethology’s proposed methodology.

## Embodying cognitive ethology

Cognitive ethology has the laudable goal of improving cognitive psychology’s ecological validity. Cognitive ethologists argue concerns about ecological validity emerge when cognitive psychology assumes invariance and control. Cognitive ethologists reject these assumptions, proposing cognitive psychologists explore variance in real-world settings to identify properties to be studied in the lab. Cognitive ethologists propose a methodology in which real-world investigations are conducted first. In this methodology, real-world and lab research can study the same phenomena using complementary methodologies.

In proposing their methodology, cognitive ethologists align themselves with embodied cognition: “Cognitive concepts cannot be properly understood without considering the fact that participants are embedded in an environment and that cognition is not independent of the environment” (Kingstone et al., 2008, p. 334). However, as we note, Kingstone’s version of embodied cognition adopts an ontology in conflict with cognitive ethology’s proposed epistemology. In particular, while cognitive ethology presumes appropriately identified cognitive processes can be studied in the lab, embodied cognition often challenges both the existence of mental representations and the relevance of controlled lab research.

In this final section, we explore the relationships between embodied cognition and cognitive ethology, and consider modifications to the latter’s proposed methodology. We believe cognitive ethology can align with embodied cognition without abandoning core assumptions, such as the existence of mental representations. However, when so aligned, cognitive ethologists must reevaluate their methodology.

We argue embodied cognition’s emphasis on sense–act processing poses serious challenges to cognitive ethology. Some embodied cognitivists are radically anti-representational (Chemero, 2000, 2009), inspired by reactions against sense–think–act processing (Brooks, 1991, 1999; Gibson, 1966, 1979). However, as we illustrate in Figure 1 (B), other embodied cognitivists acknowledge cognition requires combining sense–act and sense–think–act processing (Clark, 1997, 2007). “In most cases, at least, the emerging emphasis on the roles of body and world can be seen as complementary to the search for computational and representational understandings” (Clark, 1997, p. 149).

Clearly, cognitive ethology is better aligned with a less radical embodied cognition like Clark’s. However, the ontology of less radical embodied cognition still applies pressures on cognitive ethology’s proposed epistemology. For example, cognitive ethologists argue that real-world studies should be conducted first, which identify situations and variables to be fruitfully pursued later with lab research. Such research is seen as self-correcting:

If people begin to behave differently in the lab than in real life . . . the investigator is alerted to the fact that there is something in the laboratory that fails to capture what people really do in the real world. (Kingstone et al., 2008, p. 324)

Yet such self-correction requires tacitly assuming identical cognitive processes are studied in both settings. However, assuming so is unwarranted by *any* version of embodied cognition, unless strong identities can be established between environments in *both* the real world and the lab.

An alternative methodology, which is more consistent with embodied cognition, seeks *differences* between real-world and lab results. This approach embraces inevitable differences between real-world and lab environments. In so doing, it directs attention towards studying how (potentially identical) cognitive processes lead to radically different behaviors as environments change. Embodied cognition is rooted in investigations of how complex behaviors emerge when agents of constant sensory and motor elements are embedded in environments of growing complexity (Braitenberg, 1984; Grey Walter, 1950a, 1950b, 1951, 1963).

Studies have shown differences in gaze and attention between the lab and the real world (Gidlöf et al., 2013; Hayward et al., 2017; Risko et al., 2016). Hayward et al. (2017) investigated social attention in the lab by using a classic computer-based gaze-cueing task, and in the real world by using hidden cameras during an unscripted conversation. This allowed for comparisons of social attention engagement (i.e., looking at the face of another) and shifting (i.e., following the gaze of another) across contexts. This study showed social attention can be indexed in real-world settings, in addition to lab settings. Importantly, the data showed little common ground across the lab and the real-world contexts for both attentional engagement and shifting. In a similar vein, Gidlöf et al. (2013) showed differences between search and decision-making in the real world not revealed by previous lab-based studies. Gidlöf et al. (2013) had participants complete either a search task or a decision-making task in a grocery store with a mobile eye-tracking device. Eye movements were linked to both the environment and cognitive goals. For example, the evaluation phase in the decision-making task led to the participants’ gaze returning to their previous fixations more than the same phase in the search task—a difference not shown in other lab-based studies (Gidlöf et al., 2013), suggesting the environment plays a role in gaze behavior. These findings are likely because the gaze passively both takes in information and signals intent to others; the latter “signaling” component could be why looking behavior changes between real people and pictures (e.g., Risko et al., 2016). These studies demonstrate the necessity and value of cross-contextual work, and further highlight the dangers of assuming invariance and control.

Cognitive ethologists also view the goal of lab studies as providing subpersonal explanations of core cognitive processes first identified as being critical by real-world studies. However, lab research can have additional goals, providing good reasons for it to precede real-world investigations. For example, many of the core concepts of embodied cognition originate from postwar studies in cybernetics (Dawson, 2013; Dawson et al., 2010). Cyberneticists viewed behavior as arising from feedback relations between agents and their worlds (Ashby, 1956; Wiener, 1948); these views were crucial inspirations for early cognitivism (Miller et al., 1960). However, cyberneticists realized feedback’s dynamic, nonlinear nature was nearly impossible to study analytically with mathematical methods, particularly with real-world agents embedded in real-world environments (Ashby, 1960).

In response, cyberneticists adopted a synthetic methodology (Braitenberg, 1984; Dawson, 2004; Minsky, 1985). When following the synthetic approach, researchers begin by building simplified agents whose emergent behaviors can be studied in simplified environments. For example, Ashby (1960) recognized feedback amongst four machines defined a mathematically intractable system. Ashby understood such feedback relations by building a machine—the Homeostat—which physically realized the interactions of the four devices. “A better demonstration can be given by a machine, built so that we know its nature exactly and on which we can observe what will happen in various conditions” (Ashby, 1960, p. 99). William Grey Walter (1950a, 1950b, 1951, 1963) adopted a similar approach by studying feedback in early autonomous robots—*Machina speculatrix*.

Cyberneticists were aware of the simplified nature of synthetic models: “The Homeostat is, of course, grossly different from the brain in many respects, one of the most obvious being that while the brain has a very great number of component parts, the Homeostat has, effectively, only four” (Ashby, 1960, p. 148). However, cyberneticists did not intend their creations to model real-world agents completely. Instead, cyberneticists’ synthetic models increased their understanding of the dynamics of feedback. Grey Walter (1950b) noted of his autonomous robots:

the number of components in the device was deliberately restricted to two in order to discover what degree of complexity of behaviour and independence could be achieved with the smallest number of elements connected in a system providing the greatest number of interconnections. (p. 44)

In short, cyberneticists increased their understanding of systems by using simple, controlled synthetic models; this increased understanding could then direct investigations of more complex systems in the real world.

The synthetic methodology has several major implications for cognitive ethology. First, it demonstrates observing simpler systems can inform research closely aligned to embodied cognition. Second, it indicates the potential utility of conducting lab research prior to investigating real-world complex systems. Third, it demonstrates research can proceed by systematically varying the environment in lab settings. Of course, the synthetic methodology does not reject using real-world results to guide later lab studies. However, it reminds us real-world studies need not only involve natural observation (a method emphasized by Kingstone et al., 2008) but can also be experimental. Synthetic research is often experimental in nature; one observes how an agent’s behavior changes when the environment is systematically varied (Braitenberg, 1984; Grey Walter, 1950b).

Kingstone et al. (2008) propose cognitive ethology to alter cognitive psychology’s epistemology, improving its ecological validity. Cognitive ethology is motivated, however, by an ontological proposal: cognition is more embodied than cognitive psychology traditionally assumes. While Kingstone et al. (2008) frame cognitive ethology as being consistent with cognitive psychology’s core ontology (i.e., sense–think–act processing), we note embodied cognition often appeals to a radically different ontology (sense–act processing) and rejects cognitive psychology’s focus on mental representation. Kingstone et al. (2008) propose cognitive ethology, in part, to stimulate constructive dialogue and proposals of novel research approaches. Our contribution to this dialogue is arguing cognitive ethology must pay closer attention to which embodied approach it endorses. Radical embodied cognition adopts assumptions that work against cognitive ethology’s proposed methodology. In contrast, less radical versions of embodied cognition seem highly consistent with cognitive ethology’s aims (Risko & Gilbert, 2016). However, less radical versions also suggest how cognitive ethology can broaden its methodological proposals. For instance, synthetic methodologies show how lab studies can inform investigations of agents in real-world environments.

While our ideas emerge from exploring Kingstone’s cognitive ethology for cognitive psychology, they may also apply to variations of Griffin’s cognitive ethology for comparative psychology. Griffin (1978, 1981) introduced mental states to behaviorist theories of comparative psychology, moving them from sense–act to sense–think–act (e.g., from Figure 1 [C] to Figure 1 [A]). However, with the rise of embodied cognition, some modern theories of cognitive ethology move the discipline in the opposite direction. For instance, Barrett (2011) uses ideas from embodied cognition to argue for a comparative psychology which need not appeal to brain states or mental states, producing an approach more similar to Chemero’s (2009) radical anti-representational embodied cognitive science than Griffin’s (1978, 1981) mentalistic cognitive psychology.

We believe the rising importance of embodied cognition requires theorists to pay close attention to Simon’s (1969) parable of the ant, and to decide the degree to which psychological phenomena can be explained by appealing to bodies and environments, as well as the degree to which they can be explained by appealing to mental representations. Making such a decision places a theory on the continuum between pure sense–think–act (A) processing and pure sense–act (C) processing (see Figure 1). We have argued Kingstone’s cognitive ethology is likely best served by a less radical position which includes both types of processing (B). Clearly, similar issues arise for other kinds of cognitive ethology and likely must be faced by any theory of cognition.
